# Fecal incontinence from early pregnancy to 12 months postpartum: A longitudinal cohort study from Tuscany

**DOI:** 10.1111/aogs.70279

**Published:** 2026-06-09

**Authors:** Amerigo Ferrari, Elena Pisacreta, Manila Bonciani, Maria Magdalena Montt‐Guevara, Tommaso Simoncini, Paolo Mannella

**Affiliations:** ^1^ Division of Obstetrics and Gynecology, Department of Clinical and Experimental Medicine University of Pisa Pisa Italy; ^2^ Institute of Management, MeS (Management and Health) Laboratory Scuola Superiore Sant'Anna Pisa Italy

**Keywords:** anal incontinence, fecal incontinence, longitudinal study, patient‐reported outcomes, pelvic floor disorders, postpartum, pregnancy

## Abstract

**Introduction:**

Fecal incontinence (FI) during pregnancy and after childbirth is an underrecognized condition that may affect women's physical, psychological, and social well‐being. Evidence on its prevalence and determinants over the full perinatal period remains limited. This study aimed to assess the evolution of FI from early pregnancy to 12 months postpartum and to identify sociodemographic, clinical, obstetric, and care‐related factors associated with symptom severity and occurrence.

**Material and Methods:**

We conducted a prospective cohort study using data from a regional longitudinal maternity survey administered through the *hAPPyMamma* application in Tuscany, Italy. Women who completed all questionnaires, including the Wexner scale, between March 2019 and March 2023 were included. The time points were first and third trimesters (T0–T1), and three, six, and 12 months postpartum (T2–T4). FI prevalence and mean Wexner scores were calculated at each time point. Determinants of FI occurrence and severity were examined, respectively, through panel logistic and linear regression models adjusted for sociodemographic, clinical, obstetric, and pathway‐related variables.

**Results:**

A total of 10 576 women completed all follow‐up assessments with no missing data. FI prevalence ranged from 3.5% at T0 to 5.5% at T2, declining to 3.0% at T4. Mean Wexner scores varied only modestly across the study period, from 5.6 at T0 to 4.2 at T4, indicating overall mild but persistent symptoms throughout pregnancy and the postpartum period. Adjusted panel regression models confirmed the significant association of older age, overweight, non‐Italian nationality, lower education, lower income, high‐risk pregnancy, discomfort during pregnancy, operative delivery, perineal tears, episiotomy, higher neonatal birthweight, difficult access to services, and examinations not booked by the women themselves with FI severity or occurrence. FI symptoms were more severe at T1–T2 and more likely to occur at T1–T3 compared with T0. Pelvic floor muscle training was not protective.

**Conclusions:**

FI affects a minority of women but persists across pregnancy and postpartum, with several identifiable risk factors. Systematically integrating FI screening into antenatal and postnatal care may support earlier identification and more timely management, thus improving maternal health outcomes and strengthening continuity of care.

## Graphical abstract

In this study, a large longitudinal survey of pregnant women was conducted in Tuscany, assessing fecal incontinence from early pregnancy to 12 months postpartum using the Wexner scale. Panel regression models identify key sociodemographic, clinical, obstetric, and care pathway determinants of symptom severity and occurrence.

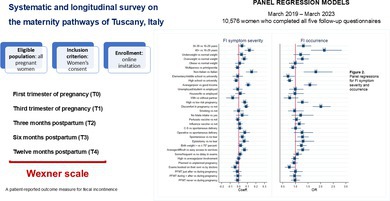

**Abbreviations**
FI: fecal incontinence
OASIS: obstetric anal sphincter injuries
PRO: patient‐reported outcome
T0: first trimester
T1: third trimester
T2: 3 months postpartum
T3: 6 months postpartum
T4: 12 months postpartum

**Key message**

Fecal incontinence affects a minority of women but can persist from early pregnancy to 1 year postpartum. Advanced age, overweight, high‐risk pregnancy, perineal trauma, operative delivery, and difficult access to services are associated with increased risk, underscoring the need for targeted assessment in vulnerable groups.

## 1 INTRODUCTION

Pregnancy‐ and childbirth‐related fecal incontinence (FI) is a clinically relevant but often underestimated condition.^1^, ^2^ Its reported prevalence varies widely depending on study design, population, and assessment tools. Cross‐sectional and longitudinal studies have shown that between 4% and 6% of women may experience FI during pregnancy, with similar or slightly higher proportions observed in the postpartum period.^3^, ^4^, ^5^ Despite these relatively modest figures compared with urinary incontinence, the functional, psychological, and social consequences of FI can be profound, significantly impairing health‐related quality of life.^6^ Yet, due to social stigma and embarrassment, many women do not disclose symptoms, and clinicians frequently omit specific screening questions.

Pregnancy itself entails several physiological and anatomical changes that may predispose women to FI. Alterations in gastrointestinal motility, increased intra‐abdominal pressure, and hormonal influences such as progesterone‐mediated smooth muscle relaxation are common, often accompanied by constipation or diarrhea, which can exacerbate or unmask continence problems.^2^, ^7^, ^8^

Several risk factors for pregnancy‐related FI have been consistently highlighted in the literature.^9^, ^10^, ^11^, ^12^, ^13^ Advanced maternal age, overweight, multiparity, spontaneous perineal tears, and operative vaginal deliveries have all been linked to a higher risk. Obstetric anal sphincter injuries (OASIS) and severe perineal lacerations represent particularly strong predictors of postpartum FI, as do prolonged second stage of labor and instrumental delivery with forceps or vacuum.^14^ Conversely, cesarean section has not consistently shown a protective effect, in contrast with what has been reported for urinary incontinence.^15^ Additional determinants include ethnicity and educational level, possibly reflecting differences in awareness, reporting, or access to health services.^5^

The impact of FI extends beyond physical symptoms. Women frequently report embarrassment, loss of confidence, anxiety, depression, and reduced participation in social and professional life. Co‐occurrence with other pelvic floor disorders such as urinary incontinence or pelvic organ prolapse may further aggravate the burden, leading to a greater reduction in quality of life than each condition alone.^6^, ^16^

Although some studies have assessed FI during pregnancy and shortly after delivery, evidence with long‐term follow‐up remains scarce. Available data suggest that while prevalence tends to decline after birth, a significant proportion of women continues to experience symptoms at 12 months or beyond, indicating that FI is not always a transient complication of pregnancy and delivery. This gap highlights the importance of prospective, population‐based studies that monitor symptoms over an extended period.

Patient‐reported outcome (PRO) measures have been increasingly recognized as fundamental for the systematic evaluation of pelvic floor disorders. They enable real‐time monitoring of symptom evolution, capture the patient's perspective, and provide real‐world evidence to inform both clinical practice and healthcare policy. The International Consortium for Health Outcomes Measurement (ICHOM) has included FI among the standard maternal health domains, recommending the use of the Wexner scale as the reference instrument.^17^ The Wexner questionnaire is widely used in clinical and research contexts, and the Italian version of this scale has been recently validated.^18^, ^19^

The present study aims to fill existing gaps by analyzing patient‐reported data from a large cohort of women followed prospectively from early pregnancy to 1 year postpartum in Tuscany, Italy. Specifically, we sought to (1) estimate the prevalence and severity of FI at different stages of pregnancy and postpartum, and (2) identify the main risk and protective factors, including sociodemographic, clinical, and care pathway attributes.

## 2 MATERIAL AND METHODS

### 2.1 Study setting, design, and data source

Italy's National Health Service guarantees universal healthcare through a decentralized structure, where each region is responsible for the delivery and quality of services.^20^ Tuscany, a central Italian region with about 3.7 million residents, manages maternity care through a Regional Health Service composed of three Local Health Authorities, 26 health districts, and about 40 hospitals. Around 22 000 deliveries occur annually in the region, with most births taking place in public facilities.

As previously described, the Tuscan Regional Health Service launched (in collaboration with our research Laboratory) a longitudinal survey in March 2019 to monitor women's experiences, health outcomes, and satisfaction throughout the maternity care pathway.^21^ The survey was integrated into the hAPPyMamma application, which also includes the digital pregnancy booklet provided to all expectant mothers in Tuscany.^22^ Women who received the pregnancy booklet (either paper or digital) were invited to participate in the online survey after giving consent.

The survey consists of eight questionnaires distributed across pregnancy and the first year postpartum. Patient‐reported outcome (PRO) measures were collected at five specific time points: first trimester (T0), third trimester (T1), 3 months postpartum (T2), 6 months postpartum (T3), and 12 months postpartum (T4). The PRO measure used in this study was the Wexner scale.^23^ Besides sociodemographic and clinical information, the survey gathers data on service utilization, satisfaction, and perceived quality of care.

### 2.2 Participants

For this study, we selected all women who completed the five follow‐up questionnaires including the Wexner scale between March 2019 and March 2023. Sociodemographic, pregnancy‐related, delivery‐related, and pathway‐related variables were collected through specific questions administered at the different time points of the survey and linked using pseudo‐anonymized identifiers.

### 2.3 Outcome measure

The primary outcome was the presence and severity of FI, assessed with the Italian version of the Wexner questionnaire. The scale includes items evaluating the frequency and type of involuntary stool loss, with total scores ranging from 0 (complete continence) to 20 (complete incontinence). We defined the binary variable “Presence of fecal incontinence” as equal to 1 whenever the Wexner score was greater than zero. We considered FI absent when the Wexner score was equal to zero. Higher Wexner scores indicated more severe symptoms.

### 2.4 Statistical analysis

For each participant and time point, we calculated the Wexner score and the presence of FI. Prevalence was reported as percentages across the five observation points. All women's baseline characteristics were categorized and compared between women reporting and not reporting FI at T4 by using the *χ* ^2^ test. To investigate risk and protective factors, we estimated panel regression models using linear regressions for Wexner scores (symptom severity) and logistic regressions for the onset of FI. All regression models were multivariable‐adjusted and included sociodemographic, clinical, obstetric, and pathway‐related covariates (such as maternal age, BMI, parity, nationality, education, income, employment status, civil status, pregnancy risk status, discomfort during pregnancy, smoking, folic acid intake, pertussis and influenza vaccination, mode of delivery, perineal trauma, neonatal birthweight, access to health services, delays in pregnancy examinations, perceived involvement in decision‐making, planned pregnancy, examination booking modality, and pelvic floor muscle training). Analyses were performed using Stata version 17.0 (StataCorp, College Station, TX, USA).

## 3 RESULTS

**FIGURE 1:**
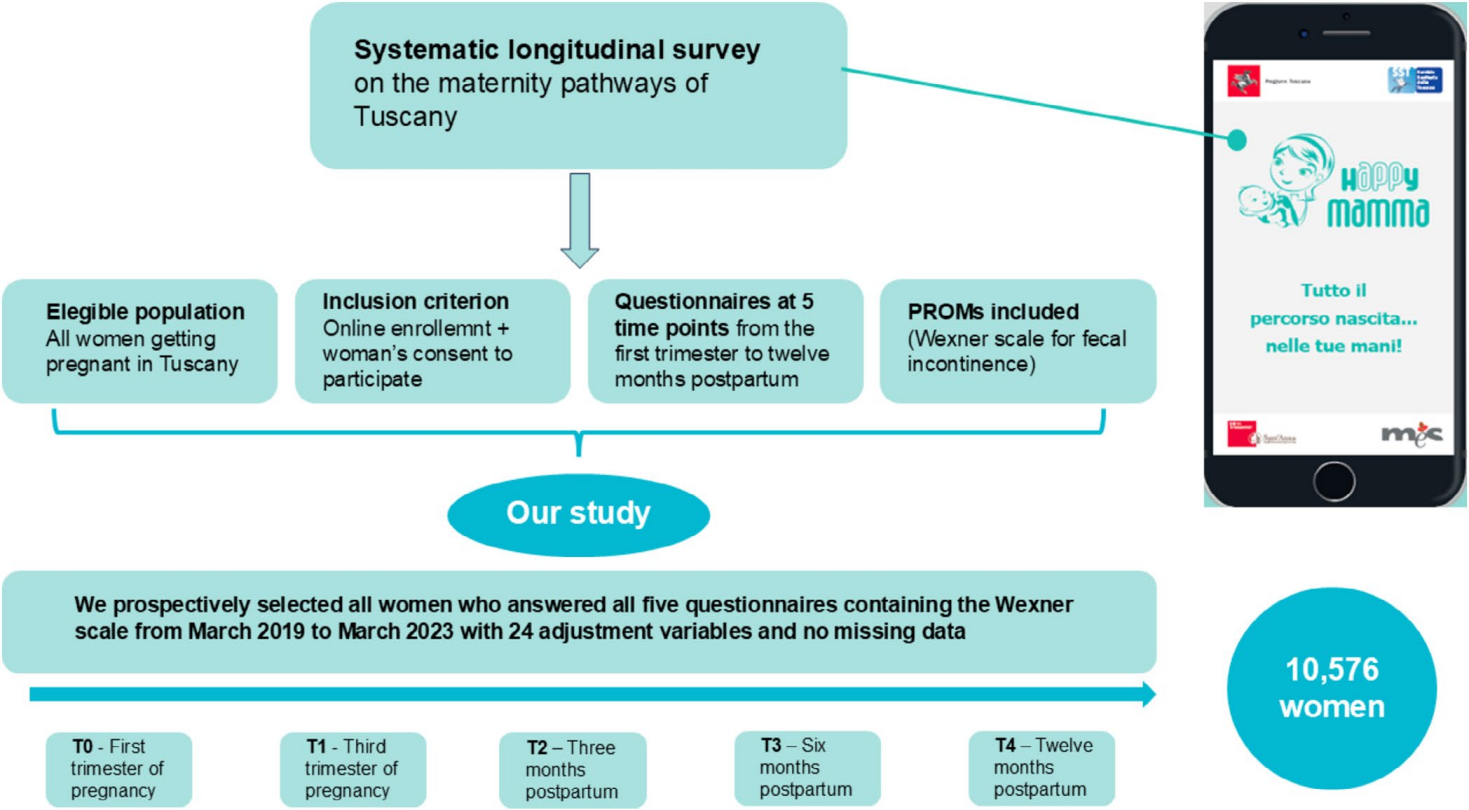
Study flowchart describing the process of cohort selection.

Women who completed all follow‐up questionnaires were 10 576 (Figure 1). The prevalence of FI was 3.5% (*n* = 366) at T0, 5.0% (*n* = 533) at T1, 5.5% (*n* = 580) at T2, 4.2% (*n* = 448) at T3, and 3.0% (*n* = 319) at T4. The mean Wexner scores (± standard deviations) were 5.6 (±3.0) at T0, 4.8 (±2.8) at T1, 5.1 (±2.9) at T2, 4.4 (±2.8) at T3, and 4.2 (±2.9) at T4 (Table 1).

**TABLE 1:** Prevalence of fecal incontinence and mean Wexner scores at each time point.

Time point	% Prevalence	Mean Wexner score (±SD)
T0 (first trimester)	3.5 (*n* = 366)	5.6 (±3.0)
T1 (third trimester)	5.0 (*n* = 533)	4.8 (±2.8)
T2 (3 months postpartum)	5.5 (*n* = 580)	5.1 (±2.9)
T3 (6 months postpartum)	4.2 (*n* = 448)	4.4 (±2.8)
T4 (12 months postpartum)	3.0 (*n* = 319)	4.2 (±2.9)

Abbreviations: *n*, number; SD, standard deviation.

Additional descriptive analyses showed that isolated gas incontinence represented only a small minority of FI cases across all time points: 1.9% at T0, 2.4% at T1, 3.4% at T2, 2.9% at T3, and 5.7% at T4 among women reporting FI. Globally, FI during pregnancy (at T0 and/or T1) was reported by 806 women (7.6%), while postpartum FI (at T2 and/or T3 and/or T4) was reported by 987 women (9.3%). Among the 806 women reporting FI during pregnancy, 234 (29.0%) continued to report symptoms postpartum, whereas 572 (71.0%) no longer reported FI after delivery. Conversely, among the 987 women with postpartum FI, 753 (76.3%) had not reported FI during pregnancy, suggesting new‐onset postpartum FI.

As given in Table 2, most respondents were between 30 and 39 years old. Compared with continent women, those experiencing FI at T4 were more frequently aged ≥40 years (14.7% vs. 11.0%), whereas women aged 16–29 years were less represented (10.3% vs. 16.8%). Maternal body mass index distribution was similar across groups, with the majority (67%–68%) of women being of normal weight. Parity differed significantly between groups, with multiparous women being more represented in the FI group (46.1% vs. 36.7%). Also, non‐Italian women reported a higher prevalence of FI at 12 months postpartum compared with Italian women (7.2% vs. 4.7%). Women with university education were more likely to report FI at T4 (63.9% vs. 55.5%). A lower proportion of women with FI at T4 reported a good income compared with those without symptoms (66.5% vs. 74.9%). Employment status and marital status did not differ significantly between groups, with most women in both groups being employed and living with a partner.

**TABLE 2:** Baseline characteristics of the study population and comparison between women with and without fecal incontinence at 12 months postpartum.

Variables	Total	No FI at T4	FI at T4	*p*‐value
	*n* = 10 576	*n* = 10 257	*n* = 319	
Age class				0.003
30–39 years	7640 (72.2%)	7401 (72.2%)	239 (74.9%)	
16–29 years	1758 (16.6%)	1725 (16.8%)	33 (10.3%)	
≥40 years	1178 (11.1%)	1131 (11.0%)	47 (14.7%)	
Maternal BMI				0.96
Normal weight	7263 (68.7%)	7048 (68.7%)	215 (67.4%)	
Underweight	762 (7.2%)	738 (7.2%)	24 (7.5%)	
Overweight	1794 (17.0%)	1737 (16.9%)	57 (17.9%)	
Obese	757 (7.2%)	734 (7.2%)	23 (7.2%)	
Parity				<0.001
Primigravida	6661 (63.0%)	6489 (63.3%)	172 (53.9%)	
Multiparous	3915 (37.0%)	3768 (36.7%)	147 (46.1%)	
Nationality				0.042
Italian	10 067 (95.2%)	9771 (95.3%)	296 (92.8%)	
Non‐Italian	509 (4.8%)	486 (4.7%)	23 (7.2%)	
Education level				0.011
University	5895 (55.7%)	5691 (55.5%)	204 (63.9%)	
Middle school or less	698 (6.6%)	680 (6.6%)	18 (5.6%)	
High school	3983 (37.7%)	3886 (37.9%)	97 (30.4%)	
Reported income				<0.001
Good	7896 (74.7%)	7684 (74.9%)	212 (66.5%)	
Average or poor	2680 (25.3%)	2573 (25.1%)	107 (33.5%)	
Employment status				0.23
Employed	9130 (86.3%)	8865 (86.4%)	265 (83.1%)	
Unemployed or student	730 (6.9%)	703 (6.9%)	27 (8.5%)	
Housewife	716 (6.8%)	689 (6.7%)	27 (8.5%)	
Civil status				0.10
Without partner	359 (3.4%)	343 (3.3%)	16 (5.0%)	
With partner	10 217 (96.6%)	9914 (96.7%)	303 (95.0%)	
High‐risk pregnancy				0.18
No	8499 (80.4%)	8252 (80.5%)	247 (77.4%)	
Yes	2077 (19.6%)	2005 (19.5%)	72 (22.6%)	
Twin pregnancy				0.77
No	10 423 (98.6%)	10 108 (98.5%)	315 (98.7%)	
Yes	153 (1.4%)	149 (1.5%)	4 (1.3%)	
Discomfort during pregnancy				<0.001
No	2921 (27.6%)	2873 (28.0%)	48 (15.0%)	
Yes	7655 (72.4%)	7384 (72.0%)	271 (85.0%)	
Smoking during pregnancy				0.62
No	9979 (94.4%)	9676 (94.3%)	303 (95.0%)	
Yes	597 (5.6%)	581 (5.7%)	16 (5.0%)	
Folate intake				0.65
Yes	10 163 (96.1%)	9858 (96.1%)	305 (95.6%)	
No	413 (3.9%)	399 (3.9%)	14 (4.4%)	
Pertussis vaccination				0.11
No	4222 (39.9%)	4081 (39.8%)	141 (44.2%)	
Yes	6354 (60.1%)	6176 (60.2%)	178 (55.8%)	
Influenza vaccination				0.60
No	8478 (80.2%)	8226 (80.2%)	252 (79.0%)	
Yes	2098 (19.8%)	2031 (19.8%)	67 (21.0%)	
Mode of delivery				0.36
Spontaneous	7117 (68.2%)	6909 (68.3%)	208 (65.6%)	
C‐Section	2590 (24.8%)	2509 (24.8%)	81 (25.6%)	
Operative	726 (7.0%)	698 (6.9%)	28 (8.8%)	
Perineal tears				0.071
No	3800 (36.9%)	3706 (37.1%)	94 (30.4%)	
Spontaneous	2988 (29.0%)	2889 (29.0%)	99 (32.0%)	
Episiotomy	908 (8.8%)	873 (8.8%)	35 (11.3%)	
C‐Section	2590 (25.2%)	2509 (25.1%)	81 (26.2%)	
Neonatal weight				0.15
Below 75° percent	7466 (72.2%)	7251 (72.4%)	215 (68.7%)	
Above 75° percent	2868 (27.8%)	2770 (27.6%)	98 (31.3%)	
Access to health services				0.017
Easy	8747 (82.7%)	8499 (82.9%)	248 (77.7%)	
Average or difficult	1829 (17.3%)	1758 (17.1%)	71 (22.3%)	
Delays in examinations due to waiting				0.008
Never	7433 (70.3%)	7230 (70.5%)	203 (63.6%)	
Sometimes or often	3143 (29.7%)	3027 (29.5%)	116 (36.4%)	
Involvement in choices				0.12
Low or average	6618 (62.6%)	6405 (62.4%)	213 (66.8%)	
High	3958 (37.4%)	3852 (37.6%)	106 (33.2%)	
Planned pregnancy				0.22
Yes	6220 (58.8%)	6043 (58.9%)	177 (55.5%)	
No	4356 (41.2%)	4214 (41.1%)	142 (44.5%)	
Examinations booking				0.52
Booked by health workers	9641 (91.2%)	9347 (91.1%)	294 (92.2%)	
Booked on their own	935 (8.8%)	910 (8.9%)	25 (7.8%)	
Pelvic floor muscle training				0.93
Just after pregnancy	1200 (11.3%)	1161 (11.3%)	39 (12.2%)	
Just before pregnancy	2354 (22.3%)	2286 (22.3%)	68 (21.3%)	
Before + after pregn.	1476 (14.0%)	1433 (14.0%)	43 (13.5%)	
Never	5546 (52.4%)	5377 (52.4%)	169 (53.0%)	

*Note*: Variables are reported as count (and percentages) in the full population and compared between women reporting and not reporting FI at T4 by using the *χ* ^2^ test.

Abbreviation: T4. 12 months postpartum.

High‐risk pregnancies were more common in women with FI (22.6% vs. 19.5%), although not significantly. Twin pregnancies were rare (~1%) and equally distributed. Women experiencing discomfort during pregnancy were more frequently in the FI group (85.0% vs. 72.0%). Mode of delivery was similar across groups, with spontaneous birth being the most frequent (65.6% in the FI groups vs. 68.3%), followed by cesarean section (25.6% vs. 24.8%) and operative delivery (8.8% vs. 6.9%). Perineal tears and episiotomy were slightly but not significantly more common among women with FI (32.0% vs. 29.0% and 11.3% vs. 8.8%, respectively), while a lower proportion had no perineal trauma (30.4% vs. 37.1%). Birthweight >3.5 kg was also more frequent in the FI group (32.0% vs. 27.7%), albeit not significantly.

Other maternal characteristics did not show marked differences. Smoking during pregnancy was reported by about 5% in both groups, and nearly all women took folic acid supplementation. Vaccination coverage was comparable, although women with FI were slightly less likely to have received pertussis vaccination (55.8% vs. 60.2%). Influenza vaccination rates were similar across groups (21.0% vs. 19.8%).

Women reporting FI at T4 found more difficulties in accessing health services (22.3% vs. 17.1%). Delays in pregnancy examinations due to long waiting times were more frequently reported by women with FI at T4 (36.4% vs. 29.5%). Perceived involvement in decision‐making did not differ substantially, with about two‐thirds of women in both groups reporting low or average involvement. Planned pregnancies were nonsignificantly less common among women with FI (55.5% vs. 58.9%). Most examinations were booked by healthcare workers in both groups. Finally, more than half of women in both groups never performed pelvic floor muscle training, while those reporting FI at T4 were slightly more likely to have carried it out after pregnancy only (12.2% vs. 11.3%).

**FIGURE 2:**
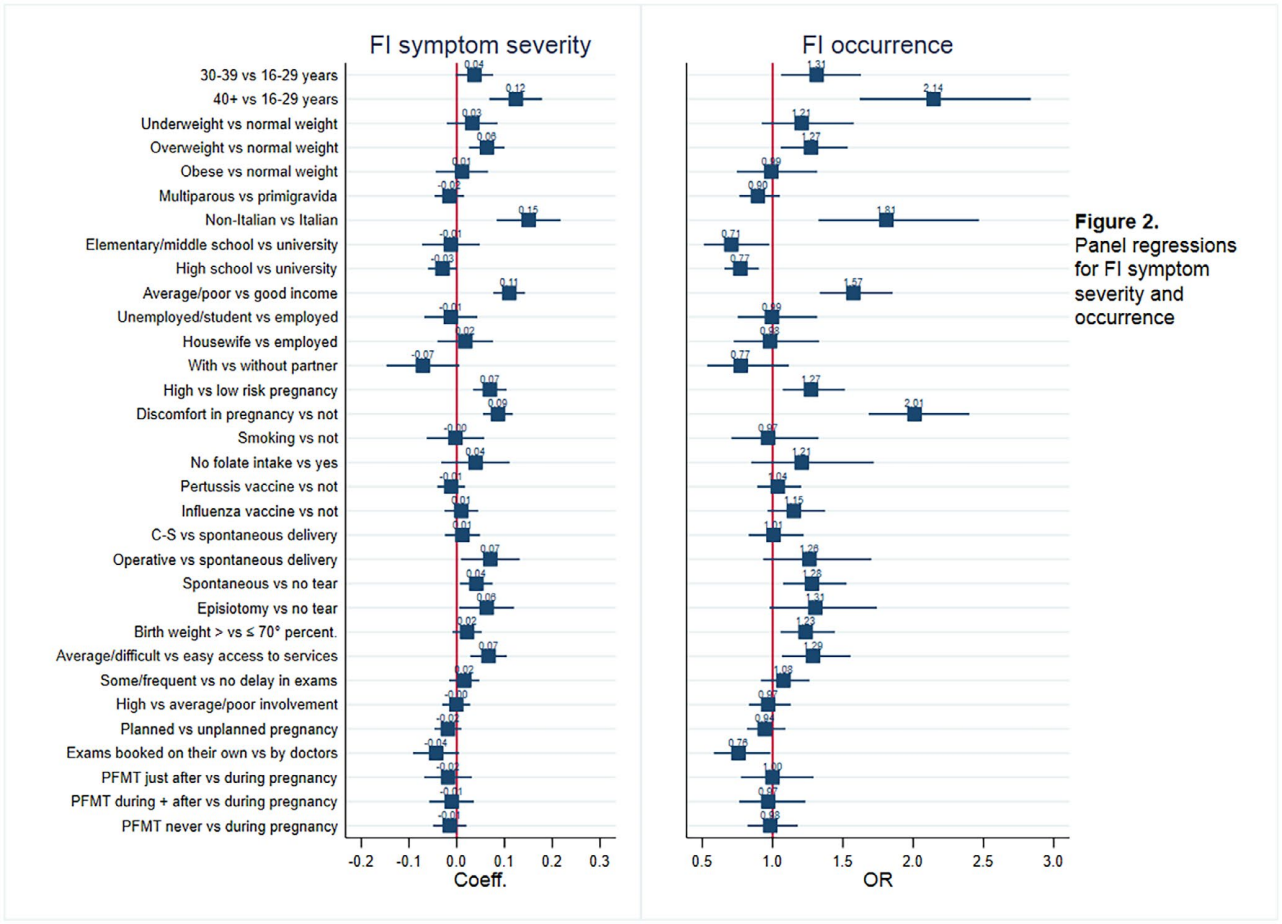
Adjusted panel regression models for fecal incontinence (FI) symptom severity and occurrence. FI prevalence and severity were assessed at five time points from the beginning of pregnancy until 12 months postpartum. The variable “time point” was included in the models but is not shown here. The value as represented on the square indicates the odds ratio (OR) or coefficient (Coeff.); the blue segment represents the 95% confidence interval; and the red line represents the baseline above or below which the variable is significant. CS, cesarean section; PFMT, pelvic floor muscle training.

Panel regression models identified several sociodemographic, clinical, and pathway‐related factors associated with FI symptom severity and occurrence at 12 months postpartum (Figure 2). Advanced maternal age was a significant determinant: women aged ≥40 years showed both more severe symptoms (coeff. 0.1, 95% CI: 0.1–0.2) and higher risk (OR 2.1, 95% CI: 1.6–2.8) compared with those aged 16–29 years, while women aged 30–39 years had not significantly greater symptom severity but were at higher risk (OR 1.3, 95% CI: 1.1–1.6). Overweight women reported greater symptom severity (coeff. 0.1, 95% CI: 0.0–0.1) and also were at higher risk of FI (OR 1.3, 95% CI: 1.1–1.5). Multiparity was not a significant risk factor.

Non‐Italian nationality was associated with greater symptom intensity (coeff. 0.2, 95% CI: 0.1–0.2) and increased risk (OR 1.8, 95% CI: 1.3–2.5). Lower education did not correlate with less severe symptoms, but FI occurrence was less likely reported by women having elementary/middle school diploma (OR 0.7, 95% CI: 0.5–1.0) or high school diploma (OR 0.8, 95% CI: 0.7–0.9). Women with average or poor income had greater symptom burden (coeff. 0.1, 95% CI: 0.1–0.1) and risk of occurrence (OR 1.6, 95% CI: 1.3–1.9). Employment and civil status were not statistically significant risk factors.

High‐risk pregnancies determined higher symptom severity (coeff. 0.1, 95% CI: 0.0–0.1) and increased risk (OR 1.3, 95% CI: 1.1–1.5). Women reporting some discomfort during pregnancy were at higher risk for both symptom severity (coeff. 0.1, 95% CI: 0.1–0.1) and FI occurrence (OR 2.0, 95% CI: 1.7–2.4). Smoking during pregnancy, folate intake, and pregnancy vaccinations were not associated with increased risk. While receiving a C‐section did not significantly affect the outcomes, operative deliveries correlated with greater symptom severity (coeff. 0.1, 95% CI: 0.0–0.1) but not increased risk. Spontaneous tears were risk factors for greater symptom perception (coeff. 0.0, 95% CI: 0.0–0.1) and FI occurrence (OR 1.3, 95% CI: 1.1–1.5), while episiotomy only linked to more severe symptoms (coeff. 0.1, 95% CI: 0.0–0.1).

Neonatal weight over 3.5 kg was a risk factor for FI occurrence (OR 1.2, 95% CI: 1.1–1.4). Women reporting moderate or high difficulty in accessing health services were at increased risk for more severe symptoms (coeff. 0.1, 95% CI: 0.0–0.1) and higher FI occurrence (OR 1.3, 95% CI: 1.1–1.6). Women who booked pregnancy exams on their own were at a lower risk of developing FI (OR 0.8, 95% CI: 0.6–1.0). Pelvic floor muscle training was not protective.

Compared with T0, women reported more severe symptoms at T1 (coeff. 0.0, 95% CI: 0.0–0.1) and T2 (coeff. 0.1, 95% CI: 0.1–0.1) and less severe symptoms at T4 (coeff. −0.1, 95% CI: −0.1 to 0.0), while there was no significant difference at T3. Finally, FI risk was higher at T1 vs. T0 (OR 1.6, 95% CI: 1.4–1.9), at T2 (OR 1.8, 95% CI: 1.5–2.1), and at T3 (OR 1.3, 95% CI: 1.1–1.5), while it was lower at T4 (OR 0.8, 95% CI: 0.7–1.0).

## 4 DISCUSSION

This prospective cohort study examined the temporal pattern of pregnancy‐related FI and identified the main sociodemographic, clinical, and pathway‐related factors associated with its occurrence and symptom severity up to 12 months postpartum. FI prevalence showed a modest increase from early to late pregnancy and remained relatively stable across the postpartum follow‐up, with values ranging from 3.5% at T0 to 5.5% at T2, followed by a gradual decline to 3.0% at T4. Mean Wexner scores followed a similar trend, with slight fluctuations over time and overall mild symptom intensity.

The descriptive analyses revealed several differences between women reporting FI at 12 months postpartum and those without symptoms. Women with FI were more frequently older, multiparous, non‐Italian, and more likely to report university education and lower income levels. Clinical and pathway‐related characteristics also differed, with a higher proportion of women reporting discomfort during pregnancy or experiencing difficult access to health services and delays in pregnancy examinations due to waiting.

Regression models confirmed the independent contribution of many of these factors, such as older age, overweight, non‐Italian nationality, lower education, and lower income status. High‐risk pregnancy and discomfort during pregnancy were also FI determinants. Several obstetric features contributed to the risk, including operative vaginal delivery, perineal tears, episiotomy, and birthweight above the 75th percentile. Among care‐related attributes, difficult access to health services and having pregnancy examinations booked by health professionals emerged as significant risk factors. Pelvic floor muscle training was not protective. More severe symptoms were reported at T1 and T2 versus T0, while FI occurrence was increased at T1, T2, and T3 compared with T0.

These findings are broadly consistent with previous evidence on pregnancy‐related FI. Earlier studies have reported FI rates of around 4% at the 3rd month of pregnancy and 5.5% at 3 months postpartum, suggesting that although only a minority of women are affected, symptoms may persist beyond delivery.^5^, ^11^, ^15^, ^24^ Our prevalence estimates fall within this range, with slightly higher values during late pregnancy and the early postpartum period and a gradual decline afterward, supporting the view that FI is often, but not always, a transient condition related to pregnancy and childbirth.

Additional longitudinal evidence reinforces this perspective: studies in women with OASIS have reported FI symptoms persisting at 24 weeks.^13^, ^14^ A recent network‐meta‐analysis further demonstrated a direct correlation between the severity of perineal laceration and long‐term anal incontinence, underscoring the etiologic relevance of perineal trauma.^25^ Long‐term follow‐up studies also show that FI may not completely resolve over time, with up to 53% of women with sphincter tears reporting anal incontinence even 5 years after childbirth.^11^ Moreover, FI prevalence around 6% at 12 months postpartum has been observed even in general obstetric populations without major trauma, indicating that persistent symptoms may also occur in low‐risk women.^4^

In addition, our results confirm several risk factors previously identified in observational studies and reviews, including advanced maternal age, overweight, high‐risk pregnancies, perineal trauma, and operative vaginal deliveries. The absence of a clear protective effect of cesarean section is likewise consistent with earlier findings. Finally, the higher likelihood of FI among highly educated and non‐native women aligns with prior work suggesting that socioeconomic and cultural factors may shape awareness, perception, and reporting of pelvic floor symptoms, contributing to higher self‐reported prevalence in these groups. Evidence on pelvic floor muscle training remains mixed: while several studies have shown that it can reduce the risk or severity of urinary and, to a lesser extent, FI in the peripartum period, its effectiveness for preventing pregnancy‐related FI specifically is still uncertain.^26^, ^27^, ^28^

This study is based on a large longitudinal dataset, with repeated observations collected from early pregnancy to 1 year after childbirth. The cohort included a substantial number of women who completed all follow‐up questionnaires, allowing us to track the evolution of FI symptoms over time with high internal consistency. Another advantage is the use of a standardized and internationally recognized PRO measure (the Wexner scale), which enabled a structured assessment of symptom severity at each time point. In addition, the regression models were adjusted for several sociodemographic, clinical, obstetric, and care‐related variables, reducing the influence of confounding and supporting the robustness of results.

Nonetheless, several limitations should be acknowledged. First, the study relies exclusively on patient‐reported data, which may be subject to misclassification, recall difficulties, or variability in the interpretation of questions. Although these limitations are intrinsic to self‐reported measures, PRO measures are increasingly recognized as valuable real‐world data sources that reflect lived experiences and are widely used to inform health system improvement.^29^, ^30^ Still, the observational design does not allow us to infer causal relationships.

Second, the cohort may not perfectly represent the broader population of pregnant women. As noted earlier, participants were more likely to be older and highly educated than the general obstetric population, which may introduce selection bias. Third, the study was conducted within a single Italian region. While the results are highly relevant for regional health system management, particularly in a decentralized national context like the Italian one, they cannot be directly generalized to the national level or to different healthcare settings.

Finally, our analysis may be affected by unmeasured confounding. We did not have access to objective clinical data that might have complemented patient self‐reports, such as detailed pelvic‐floor‐muscle training regimens, perineal ultrasound findings, perineal massages, or information on bowel disorders and infections. Not only did we lack data on the severity of perineal tears during the current childbirth, but we also did not collect information on previous OASIS, which represents one of the strongest predictors of persistent postpartum FI and may therefore have acted as an important unmeasured confounder in our analyses. Other potentially relevant variables may not have been captured by the survey.

The findings of this study have several implications for clinical practice, maternity care management, and postpartum follow‐up. Although FI affected a minority of women, its persistence throughout pregnancy and up to 12 months postpartum highlights the need to recognize FI as an important component of maternal health rather than a marginal or transient symptom. The identification of specific risk groups (such as older and overweight women, non‐Italian women, and those experiencing high‐risk pregnancies or significant discomfort during gestation) may help clinicians target early assessment and counseling to women who are more likely to develop FI.

The association between obstetric factors and FI, particularly operative vaginal delivery, perineal tears, episiotomy, and high neonatal birthweight, emphasizes the importance of careful intrapartum management and postpartum assessment. While these factors cannot always be prevented, their presence should prompt proactive monitoring after delivery, including early inquiry about bowel symptoms and the timely referral to pelvic floor specialists when needed.

Moreover, the role of care pathway attributes (such as perceived difficulty accessing services and delays in examinations) underscores that organizational aspects of maternity care can contribute to maternal health outcomes. Ensuring timely access to antenatal visits, diagnostic services, and continuity of care could mitigate part of this risk and help identify symptoms earlier. The emergence of a protective effect for women booking examinations independently also suggests that engagement in one's own care pathway may positively affect health outcomes, further reinforcing the value of patient empowerment and clear communication during pregnancy.

## 5 CONCLUSION

This study provides a comprehensive overview of pregnancy‐related FI by examining its evolution from early gestation to 1 year postpartum and identifying a wide range of individual, clinical, and organizational determinants. Although FI affected a relatively small proportion of women, several factors (including advanced maternal age, overweight, non‐Italian nationality, high‐risk pregnancies, perineal trauma, and difficulties in accessing care) were associated with increased risk or greater symptom burden. These findings underscore the need for systematic assessment of bowel symptoms during antenatal and postnatal care, with particular attention to women presenting recognized risk factors. Hence the importance of systematically including FI in antenatal and postpartum assessments, adopting a proactive and patient‐centered approach to bowel health during the perinatal period. Early detection, timely referral, and personalized management could substantially reduce the physical, emotional, and social impact of FI and ultimately improve women's quality of life, contributing to more comprehensive and equitable maternity care.

## AUTHOR CONTRIBUTIONS

Amerigo Ferrari: conceptualization, data curation, formal analysis, investigation, methodology, software, writing—original draft, writing—review and editing. Elena Pisacreta, Manila Bonciani, and Maria Magdalena Montt‐Guevara: conceptualization, data curation, investigation, methodology, writing—original draft. Tommaso Simoncini and Paolo Mannella: conceptualization, data curation, investigation, supervision, validation, writing—review and editing.

## FUNDING INFORMATION

No funding was received for this study.

## CONFLICT OF INTEREST STATEMENT

All the authors have no conflicts of interest to declare.

## ETHICS STATEMENT

The systematic maternity survey was authorized by the four regional ethics committees as follows: Area Vasta Nord Ovest (November 28, 2017), Area Vasta Centro (December 6, 2017), Area Vasta Sud Est (December 12, 2017), and Meyer Pediatric Ethics Committee (December 14, 2017).^21^ Its implementation is regulated by the Decree of the President of Tuscany Region n. 6/R/2013, in line with national guidelines on the use of personal data for healthcare satisfaction surveys. Within this framework, PRO measures are considered equivalent to other user experience surveys and do not require additional informed consent. Participants were nonetheless informed that their participation was voluntary and that they could withdraw at any stage. Data were pseudo‐anonymized.

## Data Availability

The data that support the findings of this study are available on request from the corresponding author. The data are not publicly available due to privacy or ethical restrictions.

## References

[1] Brincat C , Lewicky‐Gaupp C , Patel D , et al. Fecal incontinence in pregnancy and post partum. Int J Gynaecol Obstet. 2009;106:236‐238. doi:10.1016/j.ijgo.2009.04.018 19481750 PMC2752744

[2] Shin GH , Toto EL , Schey R . Pregnancy and postpartum bowel changes: constipation and fecal incontinence. Am J Gastroenterol. 2015;110:521‐529. doi:10.1038/ajg.2015.76 25803402

[3] Parés D , Martinez‐Franco E , Lorente N , Viguer J , Lopez‐Negre JL , Mendez JR . Prevalence of fecal incontinence in women during pregnancy: a large cross‐sectional study. Dis Colon Rectum. 2015;58:1098‐1103. doi:10.1097/DCR.0000000000000471 26445184

[4] Jansson MH , Franzén K , Tegerstedt G , Brynhildsen J , Hiyoshi A , Nilsson K . Fecal incontinence and associated pelvic floor dysfunction during and one year after the first pregnancy. Acta Obstet Gynecol Scand. 2023;102:1034‐1044. doi:10.1111/aogs.14614 37338103 PMC10378031

[5] Ferrari A , Bonciani M , Russo E , Mannella P , Simoncini T , Vainieri M . Patient‐reported outcome measures for pregnancy‐related urinary and fecal incontinence: a prospective cohort study in a large Italian population. Int J Gynaecol Obstet. 2022;159:1‐9. doi:10.1002/ijgo.14132 35122688

[6] Johannessen HH , Mørkved S , Stordahl A , Sandvik L , Wibe A . Anal incontinence and quality of life in late pregnancy: a cross‐sectional study. BJOG. 2014;121:978‐987. doi:10.1111/1471-0528.12643 24589074

[7] Solans‐Domènech M , Sánchez E , Espuña‐Pons M . Urinary and anal incontinence during pregnancy and postpartum. Obstet Gynecol. 2010;115:618‐628. doi:10.1097/aog.0b013e3181d04dff 20177295

[8] Jorge JMN , Wexner SD . Etiology and management of fecal incontinence. Dis Colon Rectum. 1993;36:77‐97. doi:10.1007/BF02050307 8416784

[9] Laine K , Skjeldestad FE , Sandvik L , Staff AC . Prevalence and risk indicators for anal incontinence among pregnant women. ISRN Obstet Gynecol. 2013;2013:1‐8. doi:10.1155/2013/947572 PMC3681258 23819058

[10] Zetterstrom JP , López A , Anzén B , Dolk A , Norman M , Mellgren A . Anal incontinence after vaginal delivery: a prospective study in primiparous women. BJOG. 1999;106:324‐330. doi:10.1111/j.1471-0528.1999.tb08269.x 10426238

[11] Pollack J , Nordenstam J , Brismar S , Lopez A , Altman D , Zetterstrom J . Anal incontinence after vaginal delivery: a five‐year prospective cohort study. Obstet Gynecol. 2004;104:1397‐1402. doi:10.1097/01.AOG.0000147597.45349.e8 15572505

[12] Hage‐Fransen MAH , Wiezer M , Otto A , et al. Pregnancy‐ and obstetric‐related risk factors for urinary incontinence, fecal incontinence, or pelvic organ prolapse later in life: a systematic review and meta‐analysis. Acta Obstet Gynecol Scand. 2021;100:373‐382. doi:10.1111/aogs.14027 33064839

[13] Richter HE , Nager CW , Burgio KL , et al. Incidence and predictors of anal incontinence after obstetric anal sphincter injury in primiparous women. Female Pelvic Med Reconstr Surg. 2015;21:182‐189. doi:10.1097/SPV.0000000000000160 25679358 PMC4481184

[14] Huebner M , Gramlich NK , Rothmund R , Nappi L , Abele H , Becker S . Fecal incontinence after obstetric anal sphincter injuries. Int J Gynecol Obstet. 2013;121:74‐77. doi:10.1016/j.ijgo.2012.10.023 23312400

[15] Larsson C , Hedberg CL , Lundgren E , Söderström L , TunÓn K , Nordin P . Anal incontinence after caesarean and vaginal delivery in Sweden: a national population‐based study. Lancet. 2019;393:1233‐1239. doi:10.1016/S0140-6736(18)32002-6 30799061

[16] Borello‐France D , Burgio KL , Richter HE , et al. Fecal and urinary incontinence in primiparous women. Obstet Gynecol. 2006;108:863‐872. doi:10.1097/01.AOG.0000232504.32589.3b 17012447

[17] Nijagal MA , Wissig S , Stowell C , et al. Standardized outcome measures for pregnancy and childbirth, an ICHOM proposal. BMC Health Serv Res. 2018;18:1‐12. doi:10.1186/s12913-018-3732-3 30537958 PMC6290550

[18] Ortenzi M , Guerrieri M , Saraceno F , et al. Prospective Italian validation of the Vaizey and Wexner and fecal incontinence severity index (FISI) questionnaires. Updat Surg. 2023;75:1617‐1623. doi:10.1007/s13304-023-01567-8 37368229

[19] Habashy E , Mahdy AE . Patient‐reported outcome measures (PROMs) in pelvic floor disorders. Curr Urol Rep. 2019;20:22. doi:10.1007/s11934-019-0888-2 30919090

[20] Ferre F , Belvis D , Iulio AG , et al. Italy: health system review. Health Syst Transit. 2014;16:1‐168. 25471543

[21] Ferrari A , Mannella P , Caputo A , Simoncini T , Bonciani M . Risk and protective factors for pregnancy‐related urinary incontinence until 1 year postpartum: a cohort study using reported outcome measures in Italy. 2023;1‐10. doi:10.1002/ijgo.15003 37462094

[22] Bonciani M , De Rosis SVM . Mobile health intervention in the maternal care pathway: protocol for the impact evaluation of hAPPyMamma. JMIR Res Protoc. 2021;10(1):e190. doi:10.2196/19073 PMC7854034 33464218

[23] Slavin V , Gamble J , Creedy DK , Fenwick J . Perinatal incontinence: psychometric evaluation of the international consultation on incontinence questionnaire—urinary incontinence short form and Wexner scale. Neurourol Urodyn. 2019;38:2209‐2223. doi:10.1002/nau.24121 31385364

[24] Everist R , Burrell M , Mallitt K‐A , Parkin K , Patton V , Karantanis E . Postpartum anal incontinence in women with and without obstetric anal sphincter injuries. Int Urogynecol J. 2020;31:2269‐2275. doi:10.1007/s00192-020-04267-8 32157322

[25] Okeahialam NA , Taithongchai A , Thakar R , Sultan AH . The incidence of anal incontinence following obstetric anal sphincter injury graded using the Sultan classification: a network meta‐analysis. Am J Obstet Gynecol. 2023;228:675‐688.e13. doi:10.1016/j.ajog.2022.11.1279 36379266

[26] Boyle R , Hay‐Smith EJC , Cody JD , Mørkved S . Pelvic floor muscle training for prevention and treatment of urinary and faecal incontinence in antenatal and postnatal women. Cochrane Database Syst Rev. 2012;10:CD007471. doi:10.1002/14651858.CD007471.pub2 23076935

[27] Sigurdardottir T , Steingrimsdottir T , Geirsson RT , Halldorsson TI , Aspelund T , Bø K . Can postpartum pelvic floor muscle training reduce urinary and anal incontinence?: an assessor‐blinded randomized controlled trial. Am J Obstet Gynecol. 2020;222:247.e1‐247.e8. doi:10.1016/j.ajog.2019.09.011 31526791

[28] Stafne SN , Salvesen KÅ , Romundstad PR , Torjusen IH , Mørkved S . Does regular exercise including pelvic floor muscle training prevent urinary and anal incontinence during pregnancy? A randomised controlled trial. BJOG. 2012;119:1270‐1280. doi:10.1111/j.1471-0528.2012.03426.x 22804796

[29] Black N . Patient reported outcome measures could help transform healthcare. BMJ. 2013;346:1‐5. doi:10.1136/bmj.f167 23358487

[30] Dawson J , Doll H , Fitzpatrick R , Jenkinson C , Carr AJ . Routine use of patient reported outcome measures in healthcare settings. BMJ. 2010;340:464‐467. doi:10.1136/bmj.c186 20083546

